# Risk factors for coagulase-negative staphylococcal surgical site infections

**DOI:** 10.1017/S0950268825100769

**Published:** 2025-11-24

**Authors:** Cynthia T. Nguyen, Amanda M. Brown, Alison K. Lew, Natasha N. Pettit, Rachel Baccile

**Affiliations:** The University of Chicago Medicine, USA

**Keywords:** methicillin resistant, prophylaxis, staphylococcus, surgical site infection, vancomycin

## Abstract

We sought to identify risk factors for coagulase-negative staphylococcal (CoNS) surgical site infection (SSI). Risk factors associated with an increased risk of CoNS SSI include male sex and asthma or COPD. Colon surgery was associated with a reduced risk of CoNS SSI.

## Introduction

Staphylococci are the most common pathogen associated with surgical site infections (SSIs), and vancomycin is commonly administered preoperatively to prevent methicillin-resistant *Staphylococcus aureus* (MRSA) SSIs [1, 2]. However, vancomycin prophylaxis may also be given due to concerns for coagulase-negative staphylococci (CoNS) SSI, which have been found to cause 7.2% of SSIs [1]. We have previously found MRSA colonization and hip and knee replacement surgeries to be risk factors associated with MRSA SSI [3]. In this analysis, we sought to identify risk factors associated with deep or organ space CoNS SSI.

## Methods

This was a case–control study performed to identify risk factors for developing a deep or organ space CoNS SSI compared to any other deep or organ space SSI. Patients who were diagnosed with a deep or organ space surgical site infection after undergoing a clean or clean-contaminated NHSN operative procedure between 1 July 2014 and 30 August 2022 were included [4]. CoNS are common skin flora, and superficial cultures isolating CoNS may not reflect true infection with this organism. Given the diagnostic uncertainty of microbiologic cultures for superficial SSIs, superficial SSIs were not included in the analysis. Cases were defined as patients with a wound culture growing CoNS, regardless of susceptibility profile (CoNS SSI), including polymicrobial cultures. Controls were defined as patients without a CoNS SSI, including patients who did not have cultures obtained or had no growth on culture. Additional methodology details, including inclusion and exclusion criteria, hospital setting, and infection prevention practices during the study period, have been previously described [3].

Identification of potential risk factors for deep or organ space CoNS SSI was initially determined by univariate analysis. We used unconditional logistic regression with Firth’s penalized likelihood to estimate crude and adjusted odds ratios (ORs) and 95% confidence intervals (CIs) and Firth’s penalized likelihood to reduce bias in estimates with small sample sizes. Variables associated with CoNS SSI with a *P*-value <0.1 in univariate analysis were candidates for multivariate modelling (selection model). The final model was selected using backward stepwise logistic regression. Goodness of fit of the final model was assessed using the AUC. A *P*-value of <0.05 was defined as statistically significant. All analyses were done in R version 4.3.2. Wound class was defined using the Centers for Disease Control and Prevention (CDC) definitions [5].

## Results

During the study period, 197 patients with a deep or organ space SSI were included in the analysis: 27 (13.8%) patients with a CoNS SSI and 170 (86%) patients without a CoNS SSI. Baseline demographics are described in Table 1. Among the 27 CoNS SSIs, 18 (67%) were polymicrobial. Susceptibility data were available for 15 (56%) of CoNS isolates and 10 were methicillin resistant.

**Table 1.** Patient and procedure characteristics

	Total *N* = 197	Patients with non-CoNS SSI *N* = 170	Patients with CoNS SSI *N* = 27	*p*-value
**Demographics**				
Age, median [IQR], and years	52 [40, 66]	52 [39.25, 65.75]	59 [42, 67]	0.413
Male	65 (33.0)	50 (29.4)	15 (55.6)	0.014
Race				0.912
Black	94 (47.7)	82 (48.2)	12 (44.4)	
White	91 (46.2)	78 (45.9)	13 (48.1)	
Other race or unknown	12 (6.1)	10 (5.9)	2 (7.4)	
Ethnicity				0.525
Not Hispanic	186 (94.4)	161 (94.7)	25 (92.6)	
Hispanic	9 (4.6)	7 (4.1)	2 (7.4)	
Unknown	2 (1.0)	2 (1.2)	0	
**Comorbidities**				
Hypertension	92 (46.7)	78 (45.9)	14 (51.9)	0.711
Active or previous smoker	86 (43.7)	70 (41.2)	16 (59.3)	0.121
Diabetes	41 (20.8)	35 (20.6)	6 (22.2)	>0.999
Asthma or COPD	36 (18.3)	26 (15.3)	10 (37.0)	0.014
BMI, median [IQR]	29.70 [25.10, 35.50]	29.45 [25.02, 35.08]	31.50 [25.40, 38]	0.415
BMI > 30	93 (47.2)	78 (45.9)	15 (55.6)	0.467
CKD on chronic HD	10 (2.3)	8 (2.3)	2 (2.2)	>0.999
Beta-lactam allergy label	42 (21.3)	36 (21.2)	6 (22.2)	>0.999
Immunocompromised	59 (29.9)	53 (31.2)	6 (22.2)	0.473
Malignancy	51 (25.9)	46 (27.1)	5 (18.5)	0.481
Receiving prednisone 10 mg >/=30 days	6 (3.0)	5 (2.9)	1 (3.7)	0.592
Primary immunodeficiency	1 (0.5)	1 (0.6)	0 (0.0)	>0.999
HIV	1 (0.5)	1 (0.6)	0 (0.0)	>0.999
Solid organ transplant	0 (0.0)	0 (0.0)	0 (0.0)	NA
**Healthcare-associated characteristics**				
Hospitalized for >72 h within 90 days prior to procedure	37 (18.8)	30 (17.6)	7 (25.9)	0.448
Hospitalized for >72 h immediately prior to inpatient procedure	25 (12.7)	22 (12.9)	3 (11.1)	>0.999
Received home wound care 30 days prior to procedure	5 (2.5)	4 (2.4)	1 (3.7)	0.525
SNF, LTAC, or nursing home resident immediately prior to procedure	10 (5.1)	8 (4.7)	2 (7.4)	0.630
Presence of at least one healthcare-associated characteristic	58 (29.4)	47 (27.6)	11 (40.7)	0.246
**Procedure characteristics**				
Index procedure				
Colorectal surgery	67 (34.0)	65 (38.2)	2 (7.4)	0.003
Abdominal or vaginal hysterectomy	38 (19.3)	35 (20.6)	3 (11.1)	0.370
Hip or knee replacement	33 (16.8)	23 (13.5)	10 (37.0)	<0.001
C-section	18 (9.1)	15 (8.8)	3 (11.1)	0.981
Spinal fusion or laminectomy	15 (7.6)	13 (7.6)	2 (7.4)	>0.999
CABG or open chest procedure	11 (5.6)	8 (4.7)	3 (11.1)	0.178
Vascular surgery	8 (4.1)	5 (2.9)	3 (11.1)	0.141
Gastric surgery	6 (3.0)	5 (2.9)	1 (3.7)	0.592
Oesophageal surgery	1 (0.5)	1 (0.6)	0 (0.0)	>0.999
Emergent procedure	26 (13.2)	20 (11.8)	6 (22.2)	0.137
Wound class [5]				0.001
I/Clean	70 (35.5)	52 (30.6)	18 (66.7)	
II/Clean-contaminated	127 (64.5)	118 (69.4)	9 (33.3)	
Procedure length, median [IQR], hours	3.28 [2.28, 4.98]	3.25 [2.31, 4.96]	3.63 [2.23, 5.23]	0.919
ASA status				0.274
1	3 (1.5)	3 (1.8)	0 (0.0)	
2	63 (32.0)	57 (33.5)	6 (22.2)	
3	112 (56.9)	96 (56.5)	16 (59.3)	
4	19 (9.6)	14 (8.2)	5 (18.5)	
Received preoperative vancomycin	49 (24.9)	36 (21.2)	13 (48.1)	0.006
Received other preoperative antibiotics	188 (95.4)	163 (95.9)	25 (92.6)	0.791
Received systemic antibiotics in the 90 days prior to surgery	70 (35.5)	58 (34.1)	12 (44.4)	0.409
Received MRSA decolonization prior to surgery	22 (11.2)	14 (8.2)	8 (29.6)	<0.001
Received antibiotic bowel preparation/decontamination prior to surgery	35 (17.8)	34 (20.0)	1 (3.7)	0.054

*Note*: Data are counted (%) unless otherwise specified. ASA, American Society of Anesthesiologists; SNF, skilled nursing facility; LTAC, long-term acute care.

By univariate logistic regression analysis, the following covariates were associated with greater odds of CoNS SSI: male sex, asthma or COPD, hip or knee replacement surgery, wound class I (vs II), receipt of preoperative vancomycin, and MRSA decolonization. Colon surgery was associated with lower odds of CoNS SSI (Table 2).

**Table 2.** Crude and adjusted odds ratios of characteristics associated with deep or organ space CoNS SSI

	Univariate			Multivariate (Selection)^a^			Multivariate (Final)^a^		
	OR	95% CI	*p*-value	OR	95% CI	*p*-value	OR	95% CI	*p*-value
Male sex	2.96	[1.31, 6.79]	0.009	3.01	[1.08, 8.98]	0.035	3.57	[1.51, 8.74]	0.004
Asthma or COPD	3.27	[1.34, 7.75]	0.010	3.46	[1.29, 9.30]	0.014	2.97	[1.16, 7.53]	0.024
Hip or knee replacement	3.77	[1.53, 9.03]	0.005	1.49	[0.44, 5.12]	0.519	–	–	–
Wound Class I (vs II)	4.4	[1.92, 10.65]	<0.001	1.23	[0.30, 4.71]	0.765	–	–	–
Receipt of preoperative vancomycin	3.43	[1.49, 7.89]	0.004	0.61	[0.16, 2.26]	0.452	–	–	–
MRSA decolonization	4.7	[1.74, 12.30]	0.003	2.29	[0.66, 8.17]	0.191	–	–	–
Colon surgery	0.16	[0.03, 0.51]	<0.001	0.21	[0.03, 0.92]	0.037	0.16	[0.03, 0.54]	0.002

a Selection model includes all variables significant on the univariate analysis. Final model includes variables from the backward stepwise regression.

In the final multivariable model, we identified two independent risk factors for CoNS SSI: male sex and having either asthma or COPD (area under the receiver operating characteristic curve [AUC], 77.69). Colon surgery was associated with a lower CoNS SSI risk (Table 2).

## Discussion

Although CoNS SSIs comprise up to 7% of SSIs, there are few publications seeking to identify risk factors for infection due to this organism. In this exploratory analysis, we found the likelihood of CoNS SSI to be higher in male patients and patients with asthma or COPD. Male patients have been identified as having higher risk of methicillin-resistant CoNS and MRSA colonization and infection, which has been attributed to poor compliance with hand-hygiene recommendations [6–9]. Though the link between of *S. aureus* colonization and infection in chronic airway diseases, such as asthma and COPD, has been previously described [10], CoNS is an unlikely respiratory pathogen, and our findings should be further explored. Additionally, we found colon surgery to be associated with a lower CoNS SSI risk likely due to the low prevalence of Staphylococci in bowel flora. By identifying high- and low-risk patients, we can better focus our SSI prevention efforts, which is crucial when resources and time are limited.

Kumagai et al. identified male sex, rheumatoid arthritis, steroid use, and severe systemic disease (American Society of Anesthesiologists Physical Status ≥3) as potential factors associated with preoperative methicillin-resistant CoNS colonization [9]. In the United States, CoNS is commonly resistant to methicillin and would not be covered with standard beta-lactam antibiotic prophylaxis (e.g., cefazolin). Kumagai et al. identified preoperative colonization with methicillin-resistant CoNS to be associated with postoperative SSIs (due to any pathogen) in patients undergoing spinal surgery [9]. In this study, all patients had a skin culture of the planned surgical site, 7 days before the procedure. Routine preoperative screening for methicillin-resistant CoNS colonization is uncommon and has several logistical hurdles. Identification of host or procedural risk factors for CoNS SSI may help guide screening in at-risk populations and antibiotic prophylaxis.

When considering the empiric addition of vancomycin to cover CoNS, the risks and benefits should be weighed. In addition to having adverse drug effects (infusion reactions, nephrotoxicity), vancomycin also comes with preprocedural logistical barriers given its weight-based dosing and prolonged infusion time. In our study, the receipt of vancomycin for preoperatively prophylaxis was associated with a reduced risk of CoNS SSI in the selection model, but not the final multivariate regression. This finding may have been due to multiple factors, including small sample size, vancomycin dose and timing, and confounding by methicillin-susceptible CoNS. It is unclear if giving vancomycin preoperatively would prevent a CoNS SSI.

Our study has several important limitations. We excluded patients with superficial SSIs; therefore, the findings may not relate to patients at risk for superficial SSIs. CoNS may cause superficial SSIs; however, given the challenge of differentiating colonization from infection upon identification on superficial cultures, these SSIs were excluded. Still, many of the CoNS SSIs had polymicrobial growth on cultures, and it is unclear whether or not the CoNS was a true pathogen in these cases.

Since CoNS are common skin colonizers, infection due to CoNS is most likely to occur when the skin is incised, at which point the exposed tissues are at risk for contamination with endogenous flora [10]. Many factors may influence this process, particularly the use of appropriate preoperative skin preparatory agents that help reduce the burden of endogenous skin flora at the site on incision [2]. Unfortunately, at our institution, documentation of these activities is unreliable, and we were unable to account for the impact of adherence to these practices.

Our study was also limited by a small sample of 27 CoNS SSIs, which limited the power of our study to only detect major contributors to increased or reduced risk of infection. Finally, we included all CoNS SSIs, including organisms that were methicillin-susceptible and those without susceptibility data. Based on our inpatient antibiogram, approximately 80% of our CoNS are methicillin resistant. Other United States hospitals may also have high methicillin-resistance rates and similarly do not perform susceptibility testing for all CoNS isolated from skin/tissue samples. Given these limitations and the exploratory nature of our study, our findings require further evaluation.

## Conclusions

We found that male patients and those with asthma or COPD had an increased risk of deep or organ space SSI due to CoNS. These findings should be further explored in order to determine how best to prevent CoNS SSIs in at-risk patients.

## Data Availability

The data are not publicly available due to sensitivity reasons but are available upon reasonable request to the corresponding author (cynthnguyen@gmail.com).
